# Reducing Glut2 throughout the body does not result in cognitive behaviour differences in aged male mice

**DOI:** 10.1186/s13104-020-05276-y

**Published:** 2020-09-16

**Authors:** Nicola Morrice, Lidy van Aalten, Alison McNeilly, Rory J. McCrimmon, Ewan R. Pearson, Rosamund Langston, Calum Sutherland

**Affiliations:** 1 Division of Cellular Medicine, School of Medicine, University of Dundee, Ninewells Hospital and Medical School, James Arnott Drive, Dundee, DD1 9SY UK; 2 Division of Systems Medicine, School of Medicine, University of Dundee, Ninewells Hospital and Medical School, James Arnott Drive, Dundee, DD1 9SY UK; 3 Division of Population Health and Genomics, School of Medicine, University of Dundee, Ninewells Hospital and Medical School, James Arnott Drive, Dundee, DD1 9SY UK

**Keywords:** Glucose, GWAS, Diabetes, GLUT2, Neuronal function

## Abstract

**Objectives:**

GLUT2 is a major facilitative glucose transporter, expressed from the *SLC2A2* gene, with essential roles in the liver. Recent work in mice has shown that preventing Glut2 production in specific neuronal populations increases sugar-seeking behaviour, highlighting the importance of *Slc2a2* gene expression in the brain. It implies that reduced GLUT2 in the brain, due to genetic polymorphisms or disease, impacts health through behaviour change. Defects in glucose transport in the brain are observed in conditions including type-2 diabetes and dementia. Few studies have directly examined the effect of modulating neuronal glucose transporter expression on cognitive function. The aim of this study was to investigate whether inactivating one *Slc2a2* allele throughout the body had major effects on cognition. Cognitive tests to assess recognition memory, spatial working memory and anxiety were performed in *Slc2a2* whole-body heterozygous mice (i.e. reduced Glut2 mRNA and protein), alongside littermates expressing normal levels of the transporter.

**Results:**

No significant effects on neurological functions and cognitive capabilities were observed in mice lacking one *Slc2a2* allele when fed a chow diet. This suggests that the minor variations in GLUT2 levels that occur in the human population are unlikely to influence behaviour and basic cognition.

## Introduction

Maintenance of glucose homeostasis is essential for survival and this is dependent on the action of cellular glucose transporters [1–5]. A major class of glucose transporters are the GLUT (glucose transporter) family, which transport glucose across the plasma membrane via facilitative diffusion [6]. GLUT2, a low affinity, high occupancy family member, is the major glucose transporter in the liver, where it is required for appropriate glucose uptake by hepatocytes, while in mice Glut2 also has an essential role in glucose-stimulated insulin secretion in the pancreas [6, 7].

Mutations in the human *SLC2A2* gene (which encodes for GLUT2 protein) cause Fanconi-Bickel syndrome (hepatomegaly and renal disease) and have been rarely found as a cause of neonatal diabetes. Genome-wide association studies indicate that *SLC2A2* sequence variation associates with risk of fasting hyperglycaemia, progression to type 2 diabetes, hypercholesterolaemia and cardiovascular diseases. We previously showed that single nucleotide polymorphisms (SNPs) found at the *SLC2A2* locus, that result in reduced *SLC2A2* gene expression, are associated with increased glycaemic response to the major diabetes therapeutic, metformin [8]. The importance of this transporter is illustrated by the fact that Glut2 null mice are not viable [6], while deletion of *Slc2a2* expression in neurons of the paraventricular nucleus results in increased sucrose-seeking behaviour in mice [9]. This latter finding suggests that Glut2 levels have a major influence on neuronal function. While most glucose transport in the brain is mediated through GLUT1 and GLUT3, there is intriguing evidence that specific neuronal populations have a reliance on Glut2 that makes them more vulnerable to pathogenic mechanisms underlying Alzheimer’s disease [10, 11]. In addition, defective glucose transport is observed in Alzheimer’s disease [12]. However, there has been limited investigation into how altered glucose transporter expression in the brain directly impacts upon behaviour and cognition. The aim of the study presented here was to examine the effects of reducing Glut2 production in the brain on behaviour and basic cognition in mice. We generated a mouse with a whole-body reduction in Glut2 (*Slc2a2*^+/−^), which models the changes in GLUT2 expression caused by common SNPs found within the human gene.

## Main text

### Animal procedures

All animal care protocols and experimental procedures were performed in accordance with the UK Home Office Animal Scientific Procedures Act (1986), the European Directive of the Protection of Animals used for Scientific Purposes 2010/ 63/ E, and with the approval of the University of Dundee Animal Ethics Committee. All personnel performing procedures are holders of UK Home Office personal licences. Work was performed under Home Office licence PE82c1898.

Slc2a2^+/−^ cryopreserved mouse sperm (C57BL/6 N-Slc2a2 < tm1b(KOMP)Wtsi > /B08, Riken reference number RBRC06334) was purchased from the Riken BioResource Center (Tsukuba, Ibaraki, Japan) and used to generate pups via in vitro- fertilisation (IVF) using C57BL/6 wild-type (WT) females (C57BL/6J, JAX™, strain code 632, bred and maintained from the original JAX strain code 000,664; this strain was introduced to Charles River Laboratories France in 1981 and UK in 2004. Breeding is in accordance with The Jackson Laboratory genetic management system. Breeding pairs were purchased from Charles River, Elphinstone, Tranent, Scotland, UK, and a colony maintained within the University of Dundee Resource Unit). Resultant Slc2a2^+/−^ animals were crossed with this same C57BL/6J WT strain to obtain Slc2a2^+/+^ (littermate controls) and Slc2a2^+/−^ pups.

For experimental procedures male *Slc2a2*^+*/*+^ (n = 8) and *Slc2a2*^+/−^ (n = 13) littermates were group housed (max 4 per cage- mixed genotype housing) and maintained at 21 °C with 12-h light/ dark cycle and had ad libitum access to water and standard chow diet (SDS Ltd, Witham, Essex, UK). Animals were weighed regularly from age 10 weeks. Behavioural tests were performed as per schematic in Additional file 1: Figure S1 in the morning of test days (before 12 pm). At week 48, animals were killed by exposure to a rising concentration of CO_2_ followed by cervical dislocation, following a 5 h fast. Fasted trunk blood sample was collected, after culling, into a lithium-heparin blood tube (BD, Wokingham, Basingstoke, England, UK), incubated at room temperature for 30 min and spun at 4 °C at 7500 rpm for 15 min. Collected blood serum was used to determine fasted insulin levels by enzyme-linked immunosorbent assay (ELISA; Crystal Chem, Elk Grove Village, IL, USA) to manufacturers’ instructions. Fasting insulin resistance index (FIRI) was calculated as standard ((fasting insulin × fasting glucose)/25) [13]. Behavioural data was captured using a Handycam HDR-CX405 camcorder (Sony, Minato City, Tokyo, Japan) and with Anymaze 6.4 software (Stoelting Co., Wood Dale, IL, USA). Data was analysed using Prism 8 (Graphpad) and are presented as mean +/− SEM and compared as indicated in relevant figure legends.

### There are no physiological differences between *Slc2a2*^+*/*+^ and *Slc2a2*^+/−^ animals maintained on chow diet

Animals were maintained on chow diet up to 48 weeks of age. No significant differences were noted in body mass between *Slc2a2*^+*/*+^ and *Slc2a2*^+/−^ groups (Additional file 1: Figures S2A and B), although there was a trend for increased weight gain in *Slc2a2*^+/−^ animals (Additional file 1: Fig. S2C; p = 0.0597, t = 2.003, df = 19). There were no significant differences between the mean fasting blood glucose (FBG) levels (Additional file 1: Figure 2D), circulating insulin levels (Additional file 1: Figure S2E) or FIRI (Additional file 1: Figure S2F) of the *Slc2a2*^+/−^ and *Slc2a2*^+*/*+^ animals at 48 weeks of age. These results show that inactivating an allele of *Slc2a2* does not affect body mass or glucose homeostasis in animals receiving chow diet.

### Inactivating one allele of *Slc2a2* does not affect spatial working memory in male mice

**Fig. 1:**
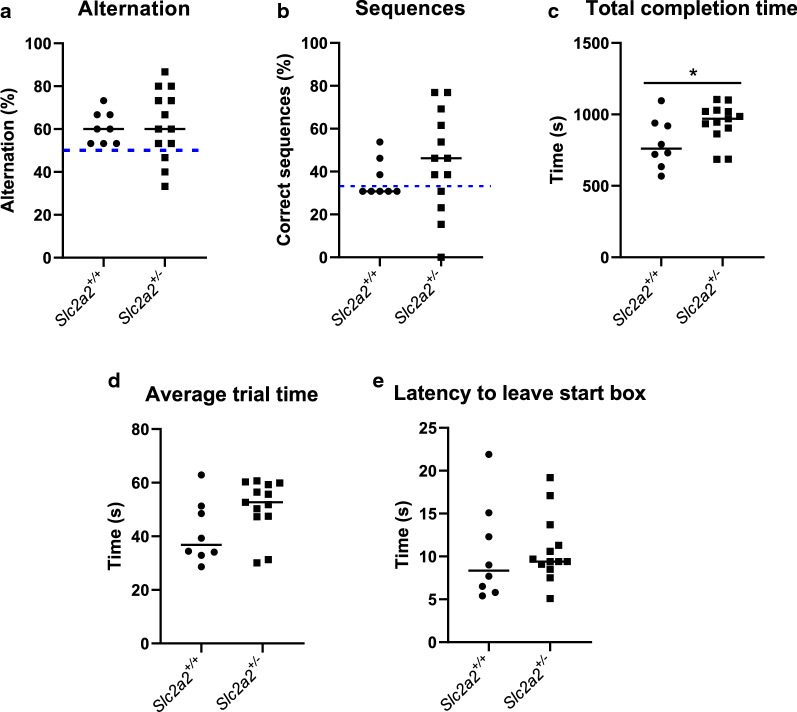
Inactivating one allele of *Slc2a2* does not affect spatial working memory in male mice. **a** Percentage alternations per group. Test performed at week 18. Blue dashed line indicates level of chance (50%). Both groups performed above level of chance: *Slc2a2*^+*/*+^ p = 0.0047, t = 4.071, df = 7 and *Slc2a2*^+/−^ p = 0.0214, t = 2.645, df = 12 (1 sample t-test). **b** Percentage LRL/ RLR sequences per group. Blue dashed line indicated level of chance (33%). Neither group performed above level of chance: *Slc2a2*^+*/*+^ p = 0.3365, t = 1.033, df = 7 and *Slc2a2*^+/−^ p = 0.1150, t = 1.700, df = 12 (1 sample t-test). **c** Total time to complete 15 trials of the test: p = 0.0472, t = 2.122, df = 19. **d** Average time per trial. E: Average latency to leave start box

To examine effects on spatial working memory, animals were tested using the T-Maze spontaneous alternation task at 18 weeks (Additional file 1: Figure S1A). Animals were allowed to spontaneously explore the maze for 15 individual trials (returning to start at the end of each test of each trial by themselves). No significant differences were observed between the two groups in the percentage alternation (Fig. 1a), which both groups performed above the level of chance (50%, as indicated by blue dashed line). Both groups performed poorly in the number of Left–Right-Left or Right-Left–Right sequences performed (Fig. 1b), which were not performed above the level of chance (33%, indicated by blue dashed line). *Slc2a2*^+/−^ animals took a significantly longer amount of time to complete 15 trials of the test (Fig. 1c), with longer mean trial time (Fig. 1d) but no differences in the latency to leave the start box during each of the 15 trials before they explored the rest of the maze (Fig. 1e). Overall, these results suggest that inactivating one allele of *Slc2a2* has no effects on spatial working memory in mice.

### Inactivating one allele of *Slc2a2* does not affect anxiety behaviour in male mice

**Fig. 2:**
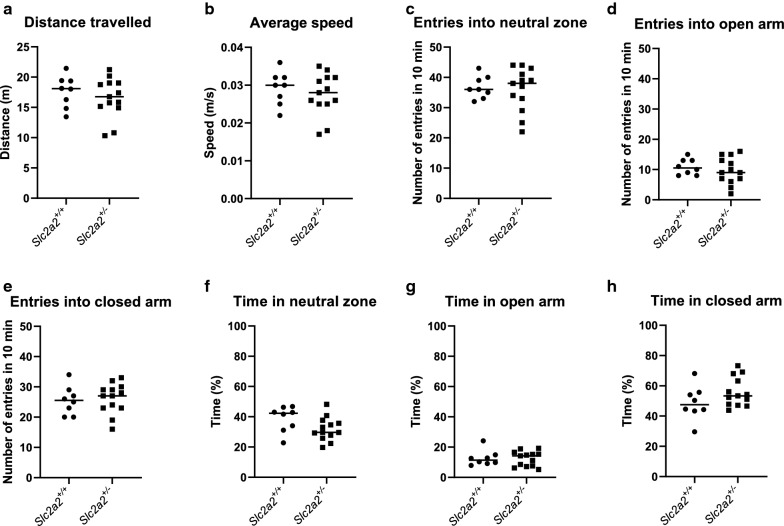
Inactivating one allele of *Slc2a2* does not affect anxiety-related behaviour in male mice. **a** Total average distance travelled in the maze over 10 min. Test performed at week 18. **b** Average speed. **c** Number of entries into neutral zone. **d** Number of entries into open arm. **e** Number of entries into closed arm. **f** Percentage time spent in neutral zone. G: Percentage time spent in open arm. **h** Percentage time spent in closed arm

To further investigate potential behaviour differences between the two groups, animals were tested with the elevated plus maze at the age of 18 weeks (Additional file 1: Figure S1B) to assess anxiety-related behaviours. No differences were observed between the two groups in any of the elevated plus maze measurements: distance travelled (Fig. 2a), average speed (Fig. 2b), number of entries into the different areas of the maze: neutral zone (Fig. 2c), open arm (Fig. 2d) and closed arm (Fig. 2e) or the percentage time spent within each zone (Fig. 2f–h). This suggests that inactivating one allele of *Slc2a2* has no effects on anxiety-related behaviours.

### Inactivating one allele of *Slc2a2* does not affect recognition memory in male mice

**Fig. 3:**
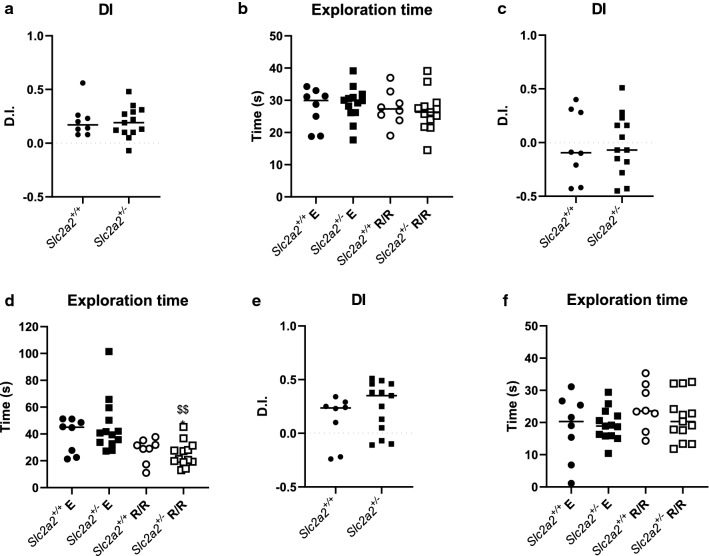
Inactivating one allele of *Slc2a2* does not affect recognition memory in mice. **a** Discrimination index (DI) of 2 min novel object recognition (NOR): average results from 2 repeats of the experiment during week 33. Both groups performed the task above the level of chance (DI = 0): *Slc2a2*^+*/*+^ p = 0.0067, t = 3.805, df = 7 and *Slc2a2*^+/−^ p = 0.004, t = 4.852, df = 12. **b** Exploration time of each group within the Encoding (E) and Retention/ Retrieval (R/R)t phases of 2 min NOR test: average results from 2 repeats of the experiment. **c** DI of 24 h NOR: results from 1 experiment during week 37. Neither group performed the task above the level of chance: *Slc2a2*^+*/*+^ p = 0.7869, t = 0.2809, df = 7 and *Slc2a2*^+/−^ p = 0.8189, t = 0.2340 and df = 12. **d** Exploration time of each group within the Encoding and Retention/ Retrieval phases of 24 h NOR: average results from 1 test. Significant differences assessed by one-way ANOVA with multiple comparisons; *: p = 0.0338 vs *Slc2a2*^+*/*+^ trial (q = 4.034; df = 38); $$: p = 0.0028 vs *Slc2a2*^+/−^ trial (q = 5.374; df = 38). E: DI of 2 min object placement test: results from 1 experiment during week 42. *Slc2a2*^+*/*+^ group did not perform the task above the level of chance: p = 0.1698, t = 1.530, df = 7. *Slc2a2*^+/−^ group did perform the task above the level of chance: p = 0.0028, t = 3.745, df = 12. F: Exploration time of each group within the Encoding and Retention/ Retrieval t phases of 2 min object placement test: results from 1 experiment

The novel object recognition (NOR) test is an established procedure to assess recognition memory [14]. Animals were assessed using both a 2 min and a 24 h NOR (as per Additional file 1: Figure S1C). No significant differences were observed during the 2 min NOR conducted at 33 weeks between *Slc2a2*^+*/*+^ and *Slc2a2*^+/−^ groups in the discrimination index (the difference in time spent exploring novel and familiar objects; DI- Fig. 3a) or in the total time spent exploring the objects in either the encoding or retention/ retrieval phases of the tests (Fig. 3b). Both groups performed the task above the level of chance (DI of 0, meaning animals spent equal amounts of time exploring objects in the retention/ retrieval phase of the task). However, both groups performed equally poorly in discriminating between objects during the 24 h NOR conducted at 37 weeks (Fig. 3c; neither group performed above the level of chance). *Slc2a2*^+/−^ animals, though, did spent less total time exploring the objects during the 10 min retention/retrieval phase of this task compared to the encoding phases (Fig. 3d). To revisit the spatial memory domain in the animals (as explored during the T-maze task), the object location test was also performed at 42 weeks (Additional file 1: Figure S1D). Again, no differences were observed between the two groups in either DI (Fig. 3e) or total exploration time measurements (Fig. 3f), however, the *Slc2a2*^+*/*+^ group did not perform the task above the level of chance and so it is difficult to draw clear conclusions from this task. Overall, these results show that inactivating one allele of *Slc2a2* has no effect on non-spatial recognition memory in mice.

### Conclusions

Most human diseases, especially chronic, age-related disease, present with minor changes in molecular pathways, which make an individual more vulnerable to environmental insults. This can be due to SNPs in key genes, that have a minor effect on the expression or function of that gene product. We have previously reported that SNPs in the GLUT2 gene (*Slc2a2*) influence response to the main drug for type 2 diabetes. Our results presented here show that minor alterations in Glut2 expression, such as those associated with specific SNPs within the human gene, are unlikely to influence “spontaneous memory and anxiety behaviours”. However, to establish the influence on response to challenges, such as poor nutrition, disease or infection, will require additional studies.

## Limitations

There are a number of limitations to the work presented in this study. Firstly, the tests were performed only on male animals [15]. Also, the animals in this study were on a regular chow diet and it is possible that effects of reduced Glut2 on cognition may be more pronounced in animals with perturbed glucose homeostasis. Future studies should include diet-induced obese animals, or animals with diabetes, to further investigate such influences. This study was designed to model the effects of common SNPs within the human population, which result in altered levels of GLUT2 expression. In this study, the removal of only one *Slc2a2* allele resulted in an approximately 30% reduction in *Slc2a2* mRNA expression (data not shown), similar to some changes in mRNA seen with specific SNPs in the human population. In contrast, the neuron-specific studies performed by Labouebe and colleagues, which resulted in changes to sucrose-seeking behaviour were performed with complete lack of Glut2 in those cells [9]. It is therefore possible that any influence of this transporter on behaviour only becomes apparent when the receptor is more dramatically perturbed. Equally, the group sizes in our study are relatively small and not powered to detect small changes. Finally, the behaviour tasks used in the Labouebe study were reward-based operant conditioning tasks, compared to the spontaneous behaviour tasks used within the study presented here, which make direct comparisons more difficult. Any future work would benefit from establishing sucrose preference to test the effects of Glut2 deficiency within hypothalamic circuits but differentiating from hippocampal circuitry by maintaining separation between food motivation and cognitive testing [16]. Glut2 expression in the brain is predominantly in the astrocytes, with only limited expression within specific neuronal populations [17]. A more comprehensive analysis of the effect of neuronal Glut2 deletion on glucose uptake and metabolism across the brain would inform a more targeted approach to dissect the role of this transporter in behaviour and cognition.

## Supplementary information

**Additional file 1.** Additional figures.

## Data Availability

The raw data for this project is available upon request through the corresponding author.

## Abbreviations

DI: Discrimination index
ELISA: Enzyme-linked immunosorbent assay
FBG: Fasting blood glucose
FIRI: Fasting insulin resistance index
IVF: *In-vitro* Fertilisation
GLUT: Glucose transporter
NOR: Novel object recognition
SNP: Single nucleotide polymorphism
