# Supplementary material for: Reducing Glut2 throughout the body does not result in cognitive behaviour differences in aged male mice
Source: BMC Res Notes. 2020 Sep 16;13:438. doi: 10.1186/s13104-020-05276-y (PMC7493158; doi:10.1186/s13104-020-05276-y)
Supplement: Supplementary file 1 — Additional file 1. Additional figures. [file 13104_2020_5276_MOESM1_ESM.pdf]

Supplementary Figure-1

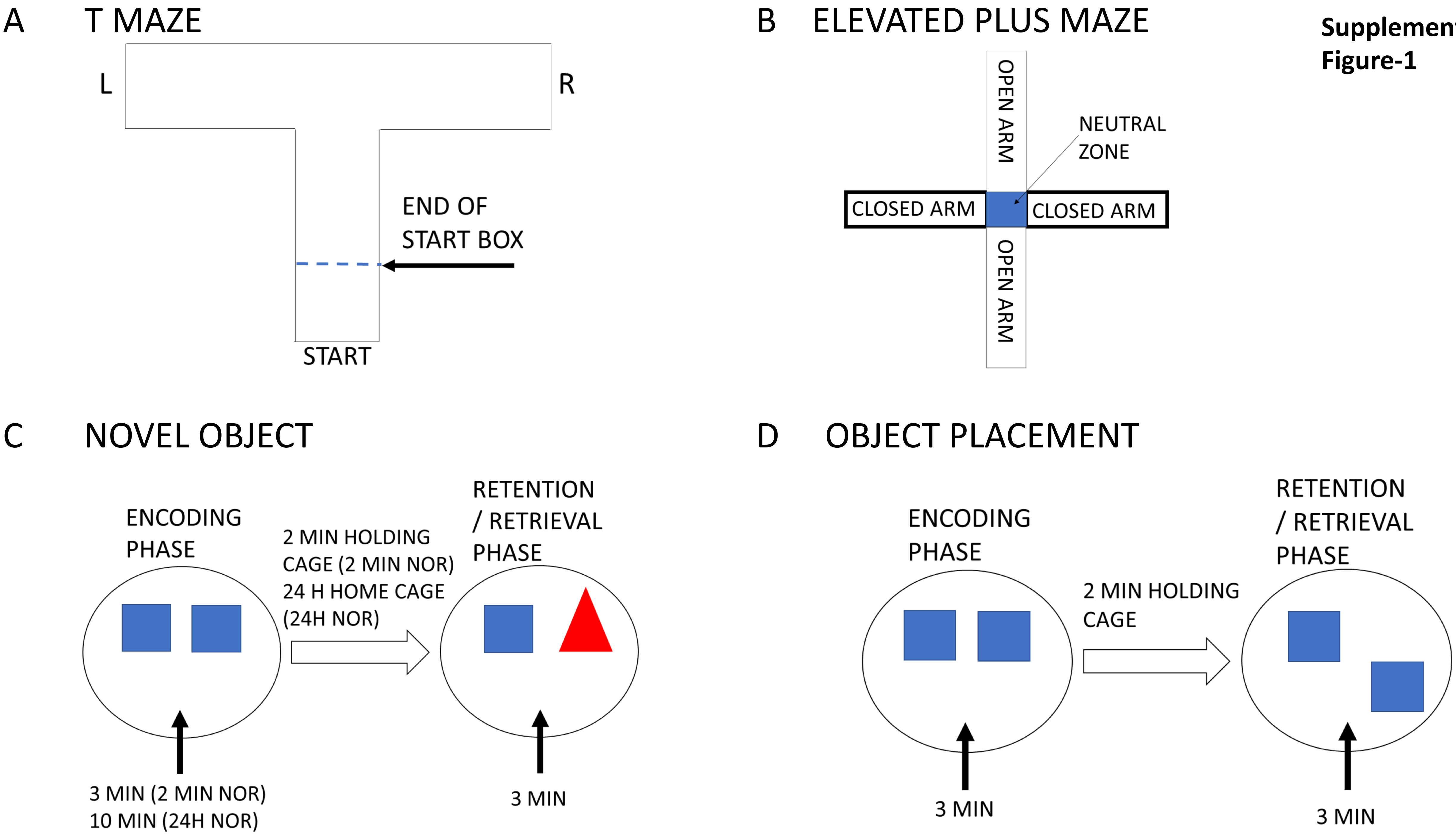

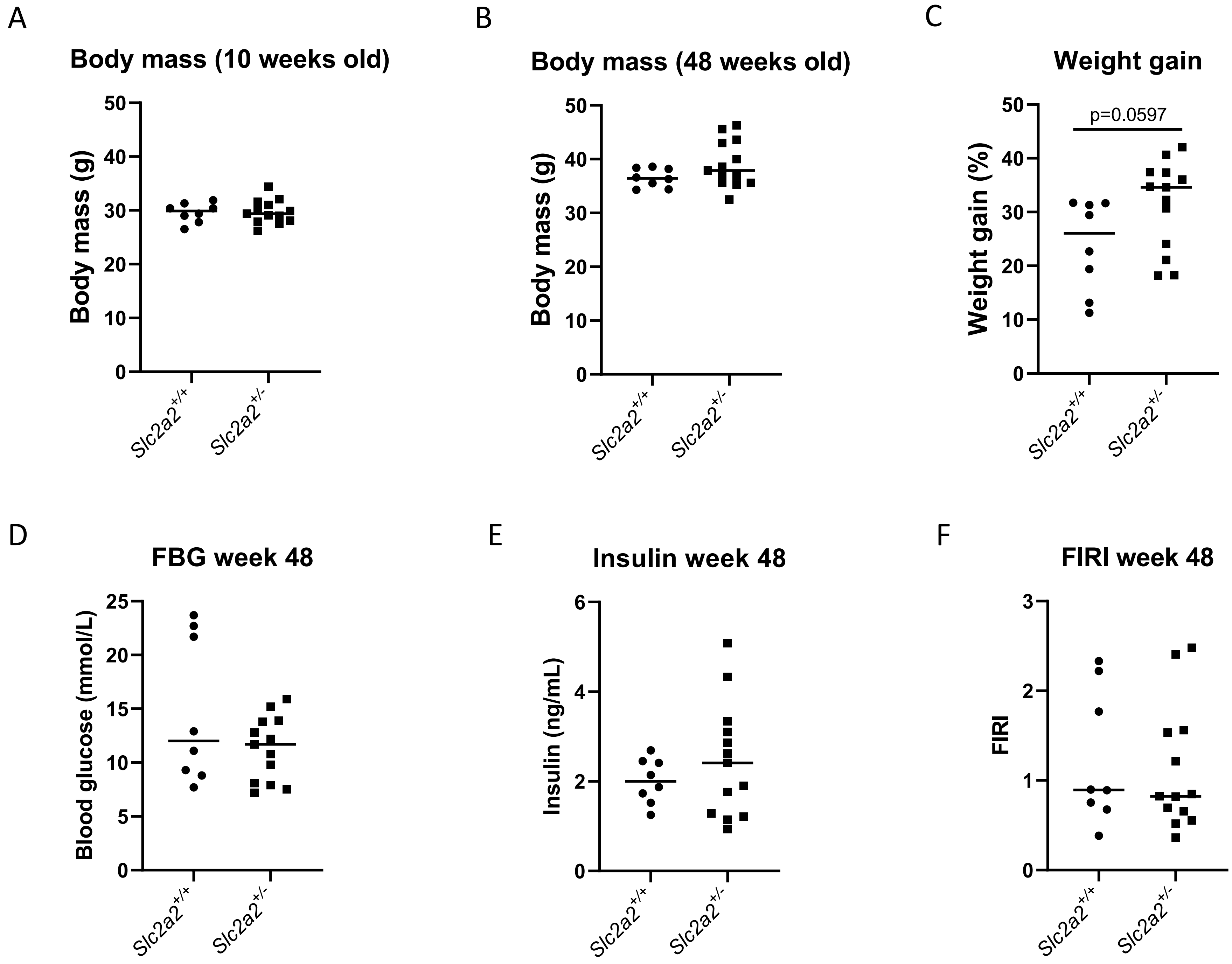

Supplementary Figure-2

A: Body mass at age 10 weeks (beginning of study; n= 8 *Glut2*$^{+/+}$ and 13 *Glut2*$^{+/-}$)
B: Body mass at age 48 weeks (end of study)
C: Body weight gain over the 38 weeks of study
D: Fasting blood glucose at the end of the study
E: Fasting circulating insulin levels at the end of study
F: FIRI at the end of study
